# Supplementary material for: Retracing and rewriting the evolutionary trajectories of mammalian developmental enhancers
Source: bioRxiv. 2026 Apr 21:2026.04.20.719714. Preprint. [Version 1] doi: 10.64898/2026.04.20.719714 (PMC13131660; doi:10.64898/2026.04.20.719714)
Supplement: Supplement 3 [file NIHPP2026.04.20.719714v1-supplement-3.pdf]

bioRxiv preprint doi: https://doi.org/10.64898/2026.04.20.719714; this version posted April 21, 2026. The copyright holder for this preprint (which was not certified by peer review) is the author/funder, who has granted bioRxiv a license to display the preprint in perpetuity. It is made available under aCC-BY 4.0 International license.

## SUPPLEMENTARY TABLES

**Table S1**: Extant and ancestral ortholog recovery per CRE.

**Table S2**: Primers and plasmids used in experiments.

bioRxiv preprint doi: https://doi.org/10.64898/2026.04.20.719714; this version posted April 21, 2026. The copyright holder for this preprint (which was not certified by peer review) is the author/funder, who has granted bioRxiv a license to display the preprint in perpetuity. It is made available under aCC-BY 4.0 International license.

## SUPPLEMENTARY FIGURES 1-11

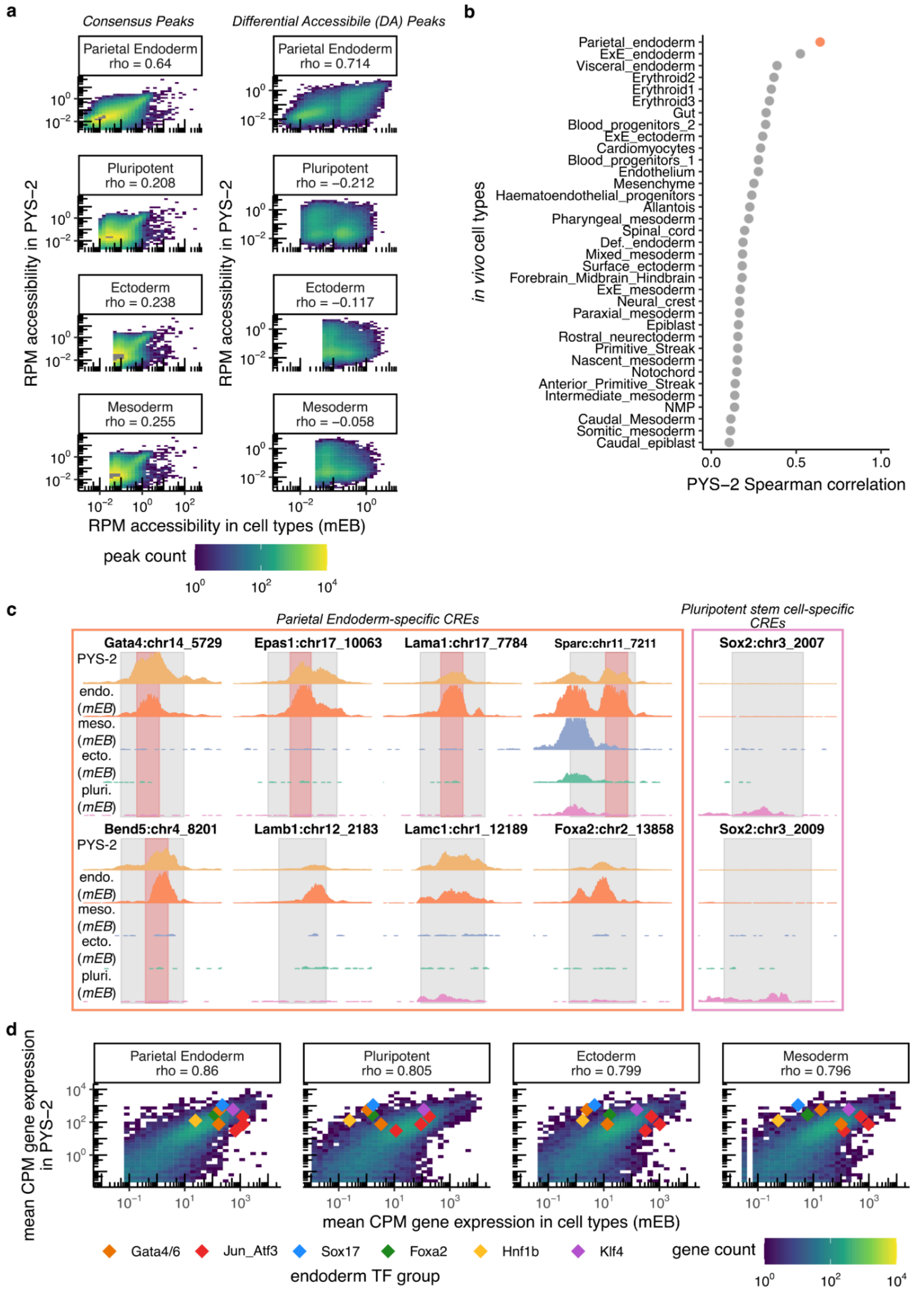


**Supplementary Figure 1. Evaluation of the PYS-2 cell line as a surrogate for the parietal endoderm lineage. a)** Comparison of normalized read counts (RPM) at consensus peaks (left) or differentially accessible peaks (right) in ATAC-seq data from mEB cell types[34] (pseudobulk; x-axes) vs. PYS-2 (bulk; y-axes). Consensus peaks are those called in both PYS-2 and mEB cells (*n* = 426,803). Differential peaks are those called as differential across mEB cell types (*n* =

bioRxiv preprint doi: https://doi.org/10.64898/2026.04.20.719714; this version posted April 21, 2026. The copyright holder for this preprint (which was not certified by peer review) is the author/funder, who has granted bioRxiv a license to display the preprint in perpetuity. It is made available under aCC-BY 4.0 International license.

56,795). Correlations (Spearman's $\rho$) are based only on peaks with RPM > 0 in both cell types being compared. **b)** Correlations (Spearman's $\rho$) for comparison of RPM at consensus peaks ($n$ = 206,973) in ATAC-seq data from *in vivo* mouse cell types[37] (pseudobulk) vs. PYS-2 (bulk). **c)** Chromatin accessibility tracks for eight parietal endoderm-specific CREs (left) and two pluripotent stem cell-specific CREs (right) validated by scQer[34], in either PYS2 cells (top track) or mEB germ layers (bottom four tracks). Shaded gray regions indicate the genomic coordinates of the original full-length CREs as validated by scQer[34] (520 bp to 1.7 kb in length). Shaded red regions correspond to coordinates of 300-bp maximum activity tile used for downstream experiments for 5 of the CREs. **d)** Comparison of mean gene expression counts (CPM) in RNA-seq data from mEB cell types (pseudobulk; x-axes) vs. PYS-2 (bulk; y-axes). Correlations (Spearman's $\rho$) are based on the log-transformed CPM expression profiles.

bioRxiv preprint doi: https://doi.org/10.64898/2026.04.20.719714; this version posted April 21, 2026. The copyright holder for this preprint (which was not certified by peer review) is the author/funder, who has granted bioRxiv a license to display the preprint in perpetuity. It is made available under aCC-BY 4.0 International license.

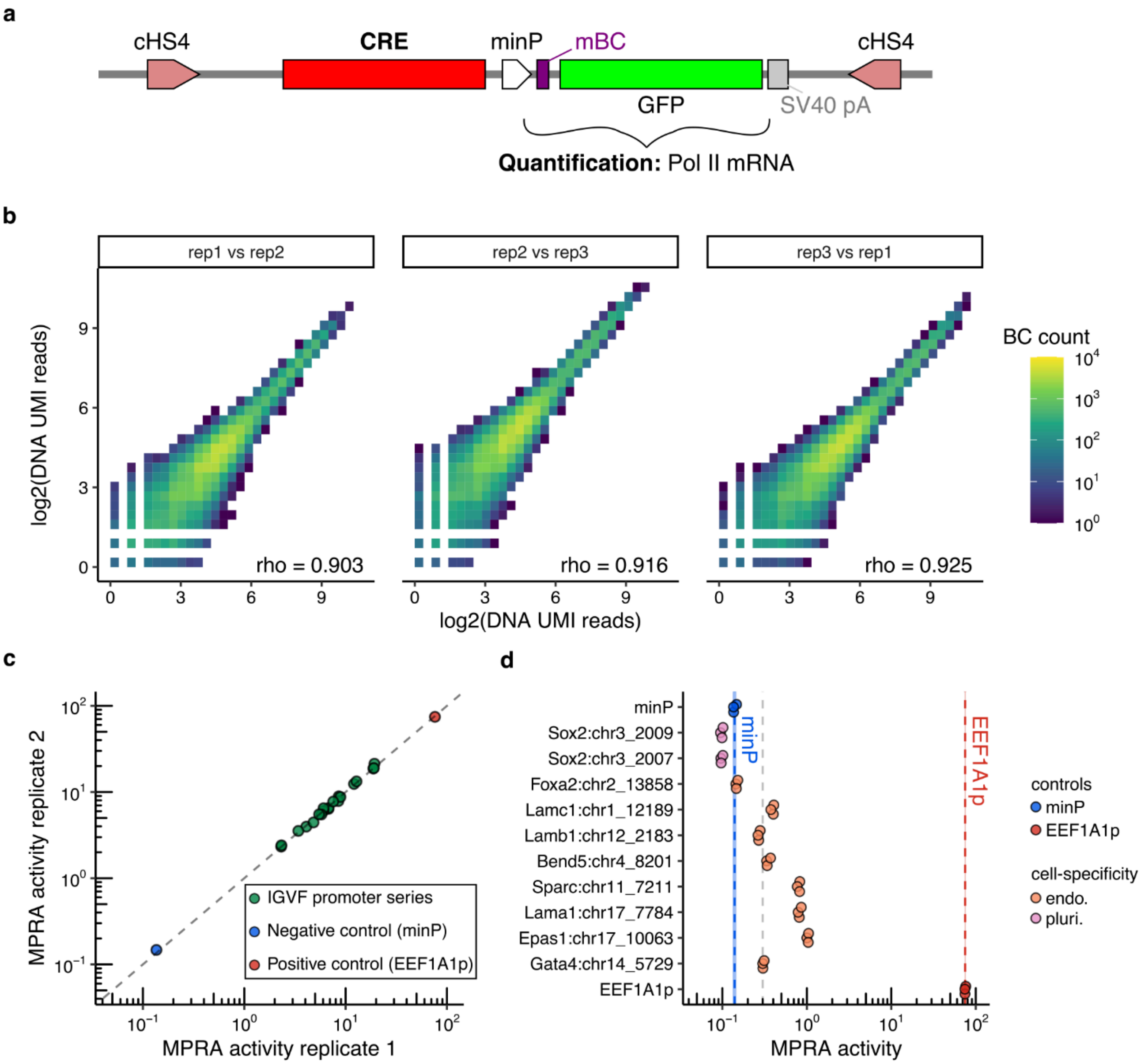


**Supplementary Figure 2**. **Quality control of MPRA-based activity profiling in PYS-2 cells**. **a)** Schematic of the MPRA reporter cassette used for transient transfection. A library of CRE is cloned to a position upstream of a minimal promoter (minP) driving a reporter gene containing a barcoded 5′ untranslated region (mBC) and a GFP open reading frame, followed by the SV40 polyadenylation signal (SV40 pA). These elements are all flanked by convergent insulators (core chicken hypersensitive site-4 from beta-globin locus, cHS4[82]). **b)** Scatterplot comparing biological replicates with respect to their log2-transformed UMI read counts for DNA-derived barcodes, each associated with a specific CRE being tested. Replicates here are independent transient transfections of the same MPRA library into PYS-2 cells (≥5 million cells transiently transfected per replicate; cells fixed 48 hours post-transfection). Points represent UMI read counts per barcode for the original parietal endoderm CREs (avg. replicate barcode recovery per element = 8781) and promoter series (avg. replicate barcode recovery per element = 105, 8799 and 4242 for IGVF, minP and EEF1A1p, respectively). Correlations (Spearman's $\rho$) were calculated by comparing the $\log_2$-transformed UMI read counts of pairs of replicates. **c)** Mean MPRA barcode activity (UMI read counts from RNA/UMI read counts from DNA) of promoters in transfection replicate 1 vs. 2. Promoters include the internal IGVF promoter series (green), minimal (minP, blue), and EEF1A1 promoter (red). **d)** MPRA activities (x-axis) of ten full-length scQer-nominated cell type-specific CREs[34], minimal promoter (minP) and EEF1A1 promoter (rows), all in PYS-2 cells. Each point represents the measured activity of a CRE in a transfection replicate. Blue dotted line and shading indicates the MPRA activity mean and standard deviation of the background control (minP), while the grey dotted line represents the 2-fold activity threshold above the background control.

bioRxiv preprint doi: https://doi.org/10.64898/2026.04.20.719714; this version posted April 21, 2026. The copyright holder for this preprint (which was not certified by peer review) is the author/funder, who has granted bioRxiv a license to display the preprint in perpetuity. It is made available under aCC-BY 4.0 International license.

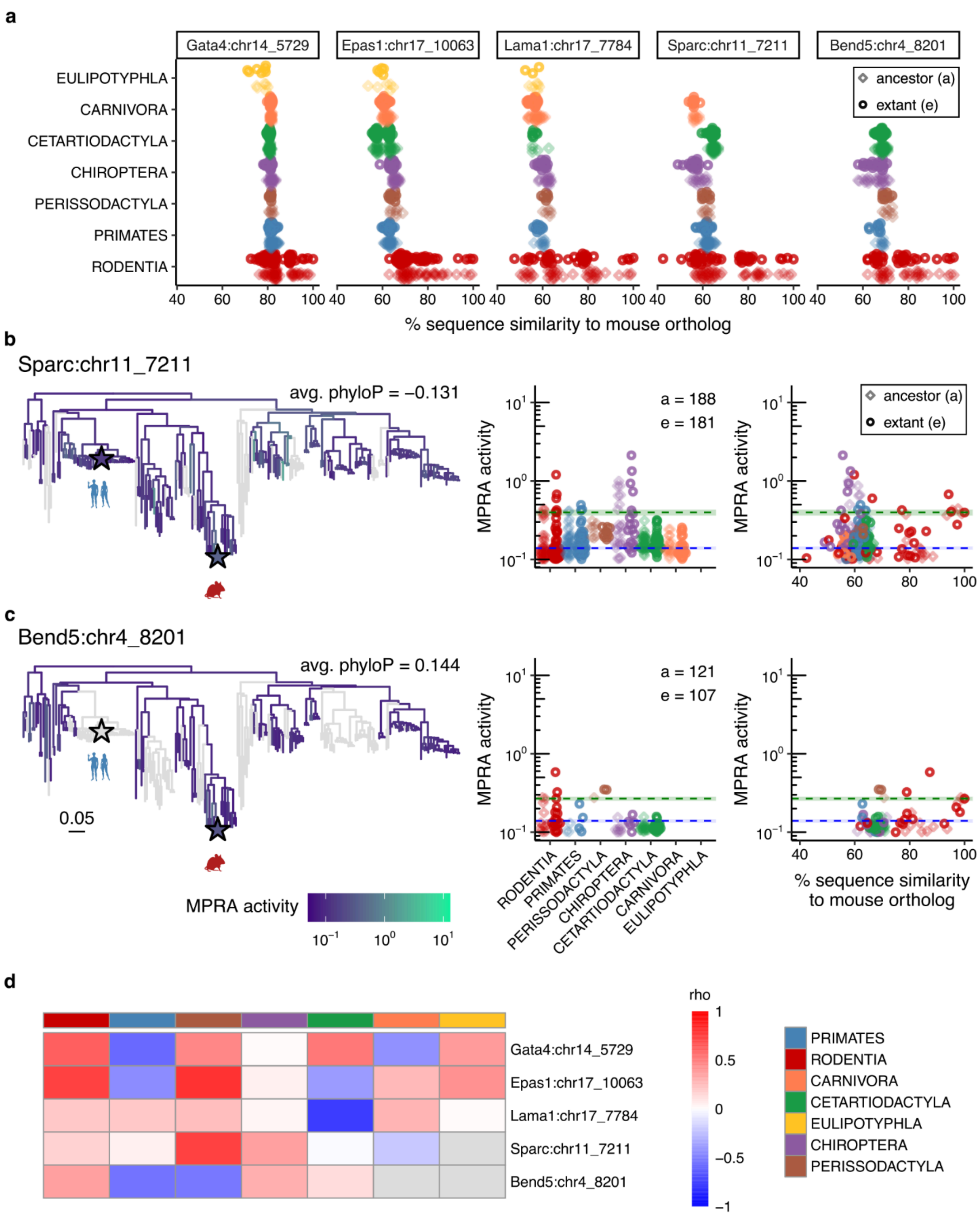


**Supplementary Figure 3. Sequence analysis of mammalian CRE orthologs. a)** Sequence identity (%) of the CRE orthologs with the endogenous mouse CRE across taxonomic orders. Lighter colors represent ancestral orthologs, and darker colors represent extant sequences. **b-c)** Left: Mammalian phylogenetic tree onto which we have projected the MPRA-measured functional activity of ancestral or extant orthologs of the *Sparc* **(b)** and *Bend5* **(c)** proximal CREs in PYS-2 cells. MPRA activities represent normalized RNA/DNA ratios. Grey branches denote species for which the ortholog was either not present in the *Cactus* alignments/reconstructions or not recovered in the assay. Colored stars denote the positions/activities of humans and mice. The mean phyloP conservation score for bases in the mouse ortholog is also shown. A scale bar at the bottom indicates branch length in units of substitution per site. Middle: MPRA activity (*y*-axes*)* broken out by taxonomic order (*x*-axes, colors). Right: MPRA activity (*y*-axes*)* as a function of sequence divergence from mouse (*x*-axes, colors). In both middle and right plots, the green and blue dotted lines correspond to activity of the mouse ortholog and minP background control, respectively. Lighter hues are used for ancestral species, and darker hues for extant species. **d)** Heatmap of correlations (Spearman's $\rho$) between MPRA measurements vs. sequence similarity to the extant mouse ortholog for each of the 5 CREs (rows), broken out by taxonomic order (columns). Grey indicates no available measurement.

bioRxiv preprint doi: https://doi.org/10.64898/2026.04.20.719714; this version posted April 21, 2026. The copyright holder for this preprint (which was not certified by peer review) is the author/funder, who has granted bioRxiv a license to display the preprint in perpetuity. It is made available under aCC-BY 4.0 International license.

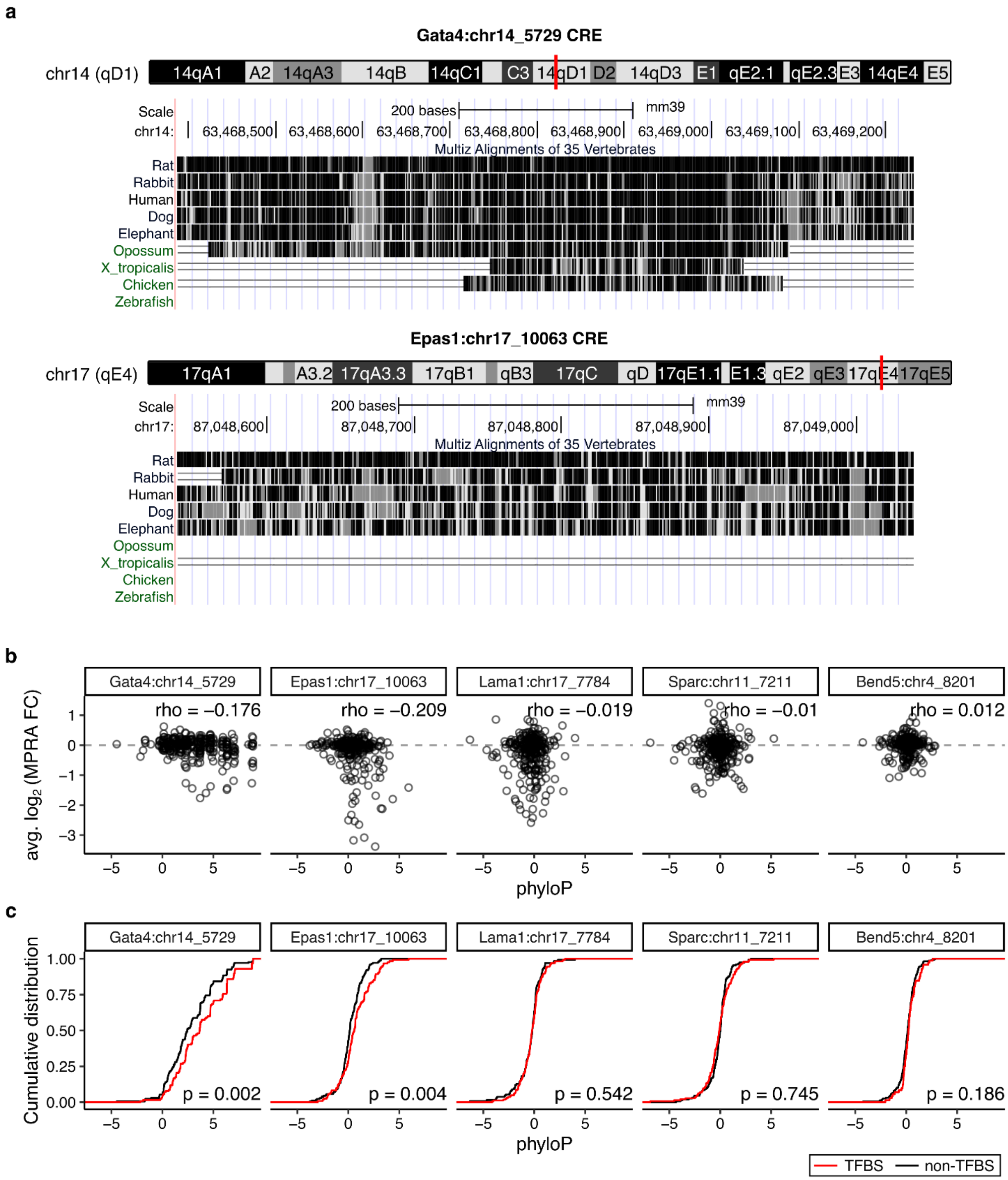


**Supplementary Figure 4. Conservation vs. saturation mutagenesis for parietal endoderm mouse CREs. a)** UCSC Multiz alignment of 60 vertebrates showing the regions containing the *Gata4* (top; chr14:63,468,388-63,469,232) and *Epas1* (bottom; chr17:87,048,296-87,049,290) CREs in the mouse genome (GRCm39/mm39). Conservation levels are indicated by shading, with darker regions corresponding to higher conservation as scored by phastCons. Placental mammals are highlighted in blue, and non-placental vertebrates in green. **b)** Scatter plot showing the lack of correlation between phyloP scores and the functional consequences of single nucleotide substitution across the CRE as measured in PYS-2 cells[38]. Correlations (Spearman's $\rho$) were calculated between phyloP scores and mean $\log_2$ fold-changes in MPRA activity. **c)** Cumulative distributions of phyloP scores (calculated from Cactus 241 mammalian species) for individual nucleotides within the CRE subsequences, comparing positions overlapping functional mouse TFBSs (red) to non-TFBS positions (black). P-values were computed to test for differences in phyloP distributions between TFBS sequences and non-TFBS sequences for each CRE using the Wilcoxon rank sum test.

bioRxiv preprint doi: https://doi.org/10.64898/2026.04.20.719714; this version posted April 21, 2026. The copyright holder for this preprint (which was not certified by peer review) is the author/funder, who has granted bioRxiv a license to display the preprint in perpetuity. It is made available under aCC-BY 4.0 International license.

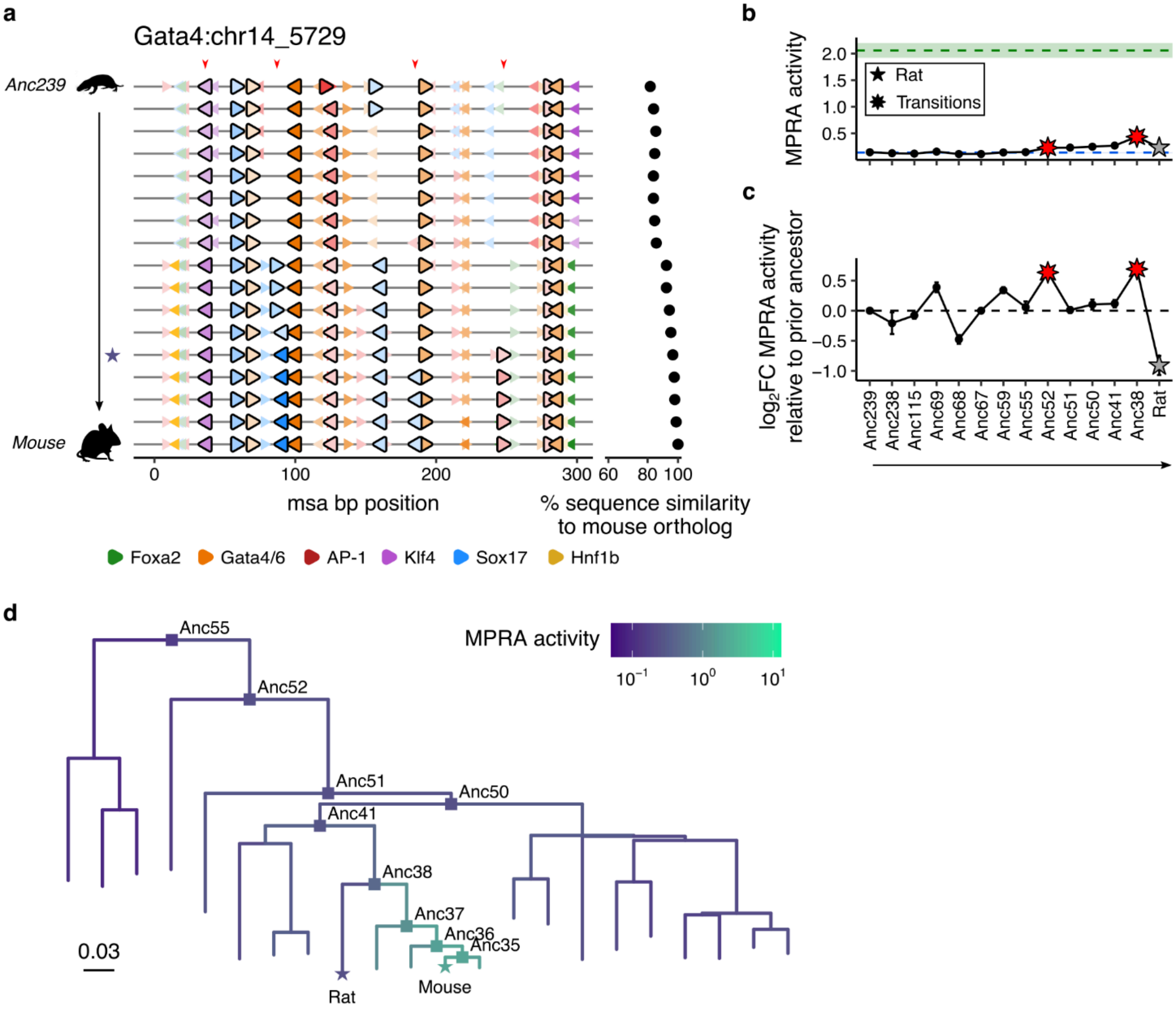


**Supplementary Figure 5. Functional evolution of the *Gata4* CRE in Rodentia. a)** Positions of predicted TFBS for each ortholog of the *Gata4* CRE along the evolutionary trajectory leading to *M. musculus*, with the subset that are functional by saturation mutagenesis MPRA assigned larger triangles with black outlines. Hue color for these predicted TFBS corresponds to predicted affinity strength, normalized across the set of sequences shown. The purple star at the left indicates the common ancestor of mouse (*M. musculus*) and rat (*R. norvegicus*). The red arrowheads at the top indicate the four TFBS that are highlighted in **Fig. 2f-g** and discussed in the main text. At the right, % sequence similarity of each ancestral ortholog to the extant mouse ortholog is shown. **b-c)** Change in MPRA activity profile (top) and the $\log_2$ fold-changes in MPRA activity relative to the immediately prior ancestor (bottom) along the evolutionary trajectory of the *Gata4* CRE leading to *R. norvegicus*. Error bars indicate the standard error across three experimental replicates. **d)** Same as **Fig. 1c**, but zooming in on a Rodentia subclade rooted at Anc55 and colored by the measured MPRA activity of *Gata4* CRE orthologs. Squares highlight ancestral nodes along the mouse and rat lineage. Stars indicate extant mouse and rat. A scale bar at the bottom indicates branch length in units of substitution per site.

bioRxiv preprint doi: https://doi.org/10.64898/2026.04.20.719714; this version posted April 21, 2026. The copyright holder for this preprint (which was not certified by peer review) is the author/funder, who has granted bioRxiv a license to display the preprint in perpetuity. It is made available under aCC-BY 4.0 International license.

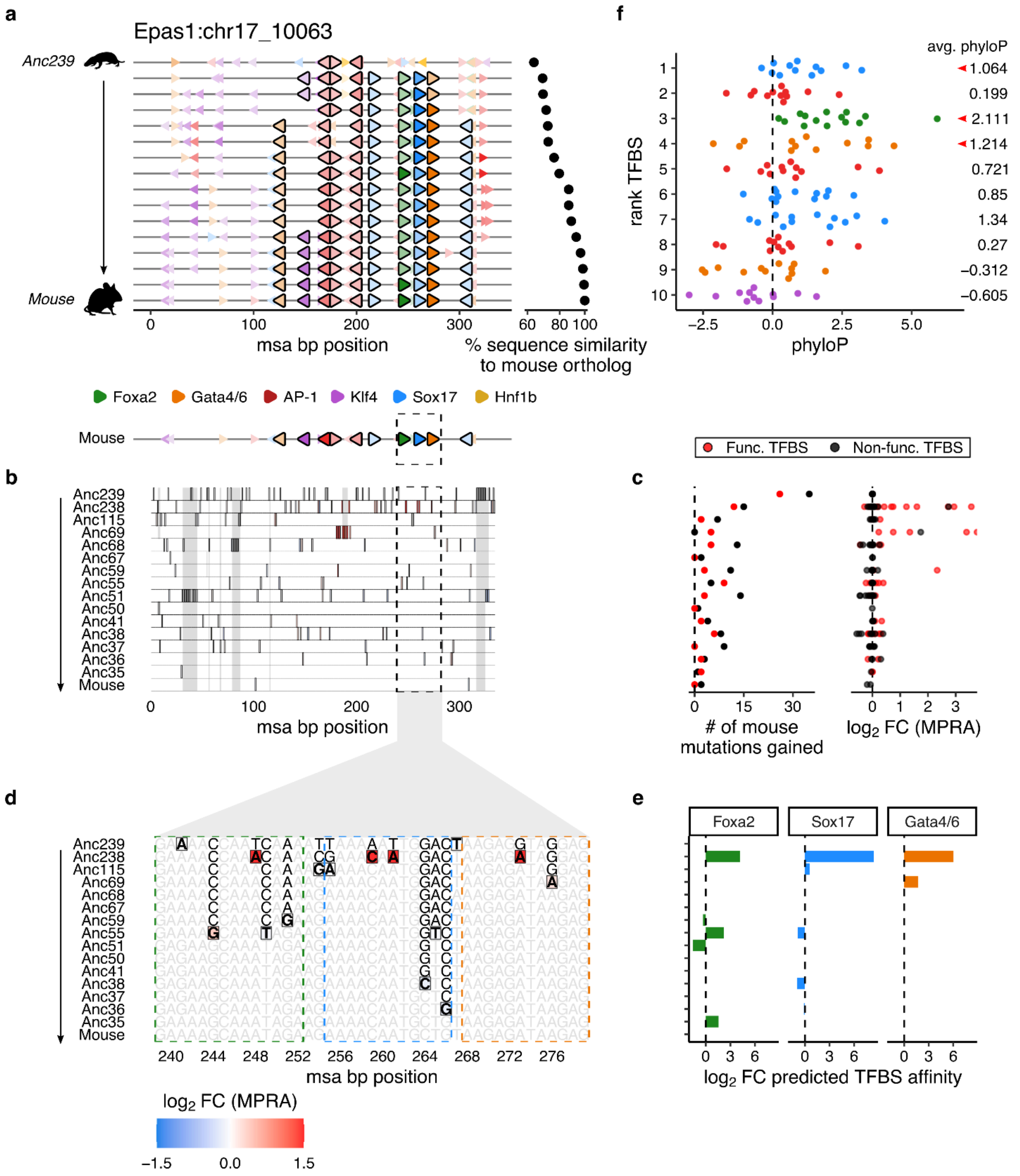


**Supplementary Figure 6**. **Evolution of a conserved *cis*-regulatory module in the *Epas1* CRE. a)** Predicted TFBS positions for each ortholog of the *Epas1* CRE along the evolutionary path from Anc239 to *M. musculus*. TFBSs that are functional by saturation-mutagenesis MPRA are indicated by larger triangles with black outlines. Hues correspond to predicted TFBS affinities, normalized across the set of sequences shown. At right, the percent sequence similarity to the extant mouse ortholog is plotted. **b)** MSA of all *Epas1* CRE sequences along the evolutionary path from Anc239 to *M.*

bioRxiv preprint doi: https://doi.org/10.64898/2026.04.20.719714; this version posted April 21, 2026. The copyright holder for this preprint (which was not certified by peer review) is the author/funder, who has granted bioRxiv a license to display the preprint in perpetuity. It is made available under aCC-BY 4.0 International license.

*musculus*. At top, predicted TFBS positions for the extant mouse ortholog are shown. Black-bordered tiles correspond to derived mutations relative to the immediately prior ancestor and are colored by $\log_2$ fold-changes in saturation-mutagenesis MPRA activity[38] (color scale at bottom left of figure; measured in mouse background). Gray columns mark derived indels not assayed by MPRA. Black dashed box denotes the conserved triplet module, mutations within which are further detailed in panels **d**-**e**. **c)** Number (left) and distribution of $\log_2$ fold-changes in MPRA activity (right) for derived mouse mutations appearing at each step along the evolutionary path to the *M. musculus Epas1* CRE, partitioned by whether mutations lie within (red) or outside (black) a functional mouse TFBS. **d)** Nucleotide-resolution view of subregion of MSA shown in panel **b**, highlighting mutations within the conserved triplet module. Black-bordered tiles correspond to derived mutations relative to the immediately prior ancestor and are colored by $\log_2$ fold-changes in saturation-mutagenesis MPRA activity[38] (color scale at bottom left of figure; measured in mouse background). Gray columns mark derived indels not assayed by MPRA. Colored dashed boxes show the positions of the three TFBS—Foxa2 (green), Sox17 (cyan), and Gata4/6 (orange)—that comprise the conserved module. **e)** $\text{Log}_2$ fold-change in predicted TFBS affinity across evolutionary steps for the three conserved TFBSs highlighted in panel **d**. Key mutations from Anc239 → Anc238 include: T→A (pos 248), associated with an 18-fold gain in predicted Foxa2 affinity and 6.5-fold gain in measured MPRA activity; A→C (pos 259) and T→A (pos 261), associated with a 338-fold gain in predicted Sox17 affinity (together) and 7.5- and 12-fold gains in measured MPRA activity (respectively); and G→A (pos 273), associated with a 65-fold increase in predicted Gata4/6 affinity and 3.0-fold gain in measured MPRA activity. **f)** PhyloP scores for the 10 functional mouse TFBSs identified by a saturation mutagenesis MPRA of the *Epas1*:chr17_10063 CRE. Each row represents a functional TFBS ranked by the magnitude of its disruption on enhancer activity (top = highest). Each point denotes the phyloP score of one base-pair within the TFBS represented on that row. At right, the mean phyloP score for each TFBS is listed; red arrows denote the three TFBSs comprising the conserved heterotypic triplet module.

bioRxiv preprint doi: https://doi.org/10.64898/2026.04.20.719714; this version posted April 21, 2026. The copyright holder for this preprint (which was not certified by peer review) is the author/funder, who has granted bioRxiv a license to display the preprint in perpetuity. It is made available under aCC-BY 4.0 International license.

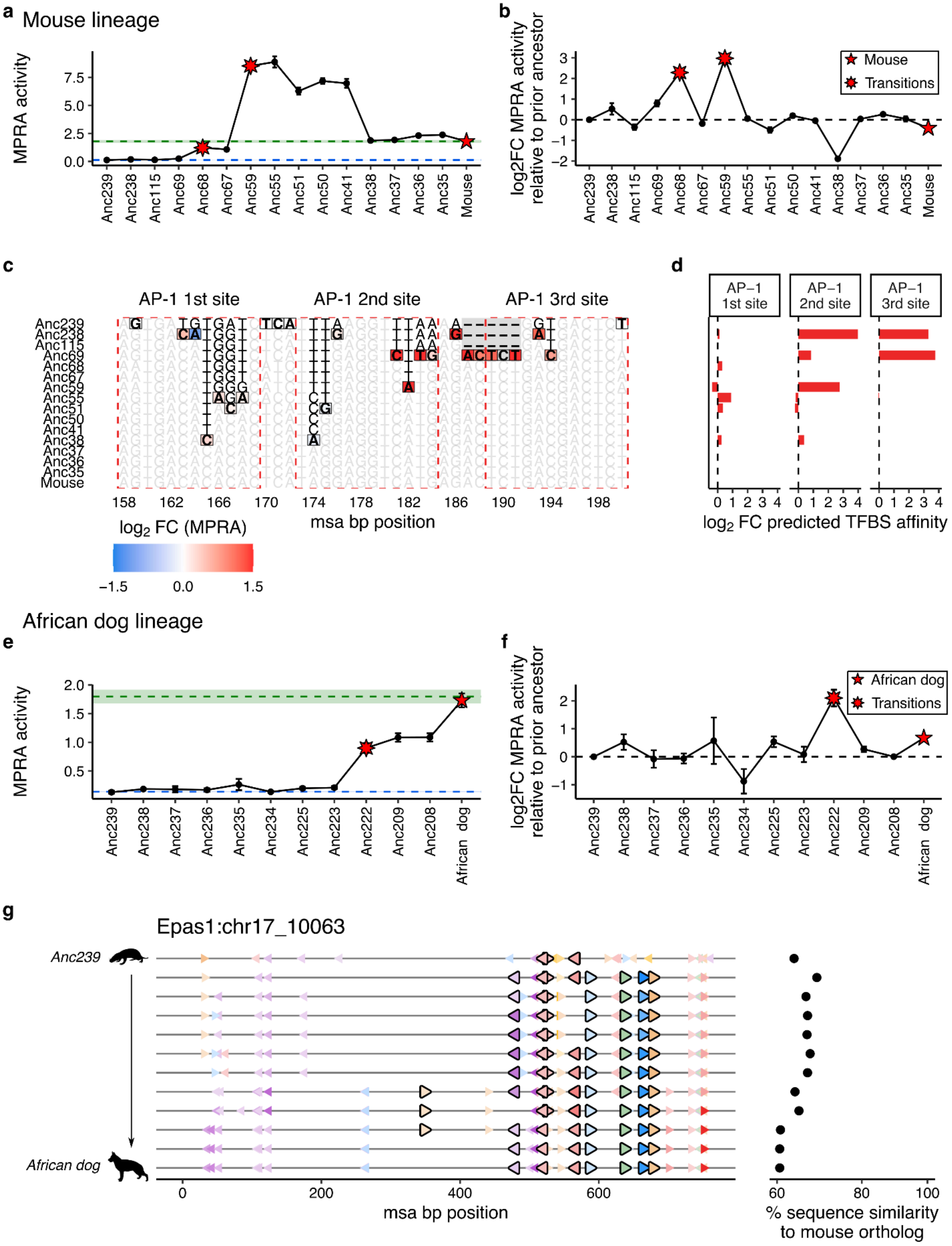


**Supplementary Figure 7. Evolution of species-specific AP-1 sites in mouse and African dog *Epas1* CRE ortholog. a–b)** MPRA activity profiles (left) and $\log_2$ fold-changes in MPRA activity relative to the immediately prior ancestor (right) of the *Epas1* CRE along the evolutionary path from Anc239 to *M. musculus*. Error bars indicate the standard error across three biological replicates. MSA of all *Epas1* CRE sequences along the evolutionary path from Anc239 to *M. musculus*. **c)**

bioRxiv preprint doi: https://doi.org/10.64898/2026.04.20.719714; this version posted April 21, 2026. The copyright holder for this preprint (which was not certified by peer review) is the author/funder, who has granted bioRxiv a license to display the preprint in perpetuity. It is made available under aCC-BY 4.0 International license.

Nucleotide-resolution view of a subregion of MSA shown in **Fig. 4a** corresponding to a portion of the homotypic AP-1 module. Black-bordered tiles correspond to derived mutations relative to the immediately prior ancestor and are colored by $\log_2$ fold-changes in saturation-mutagenesis MPRA activity[38] (color scale at bottom left; measured in mouse background). Gray columns mark derived indels not assayed by MPRA. Dashed red boxes show the positions of the three AP-1 TFBS that comprise the homotypic cluster. **d)** $\text{Log}_2$ fold-change in predicted TFBS affinity across evolutionary steps for the three AP-1 TFBSs highlighted in panel **c**, with matching rows as well. **e–f)** MPRA activity profiles (left) and $\log_2$ fold-changes in MPRA activity relative to the immediately prior ancestor (right) of the *Epas1* CRE along the evolutionary path from Anc239 to *L. pictus* (African wild dog). Error bars indicate the standard error across three biological replicates. **g)** Predicted TFBS positions for each ortholog of the *Epas1* CRE along the evolutionary path from Anc239 to *L. pictus* (African wild dog). TFBSs that are functional by saturation-mutagenesis MPRA are indicated by larger triangles with black outlines. Hues correspond to predicted TFBS affinities, normalized across the set of sequences shown. At right, the percent sequence similarity to the extant mouse ortholog is plotted.

bioRxiv preprint doi: https://doi.org/10.64898/2026.04.20.719714; this version posted April 21, 2026. The copyright holder for this preprint (which was not certified by peer review) is the author/funder, who has granted bioRxiv a license to display the preprint in perpetuity. It is made available under aCC-BY 4.0 International license.

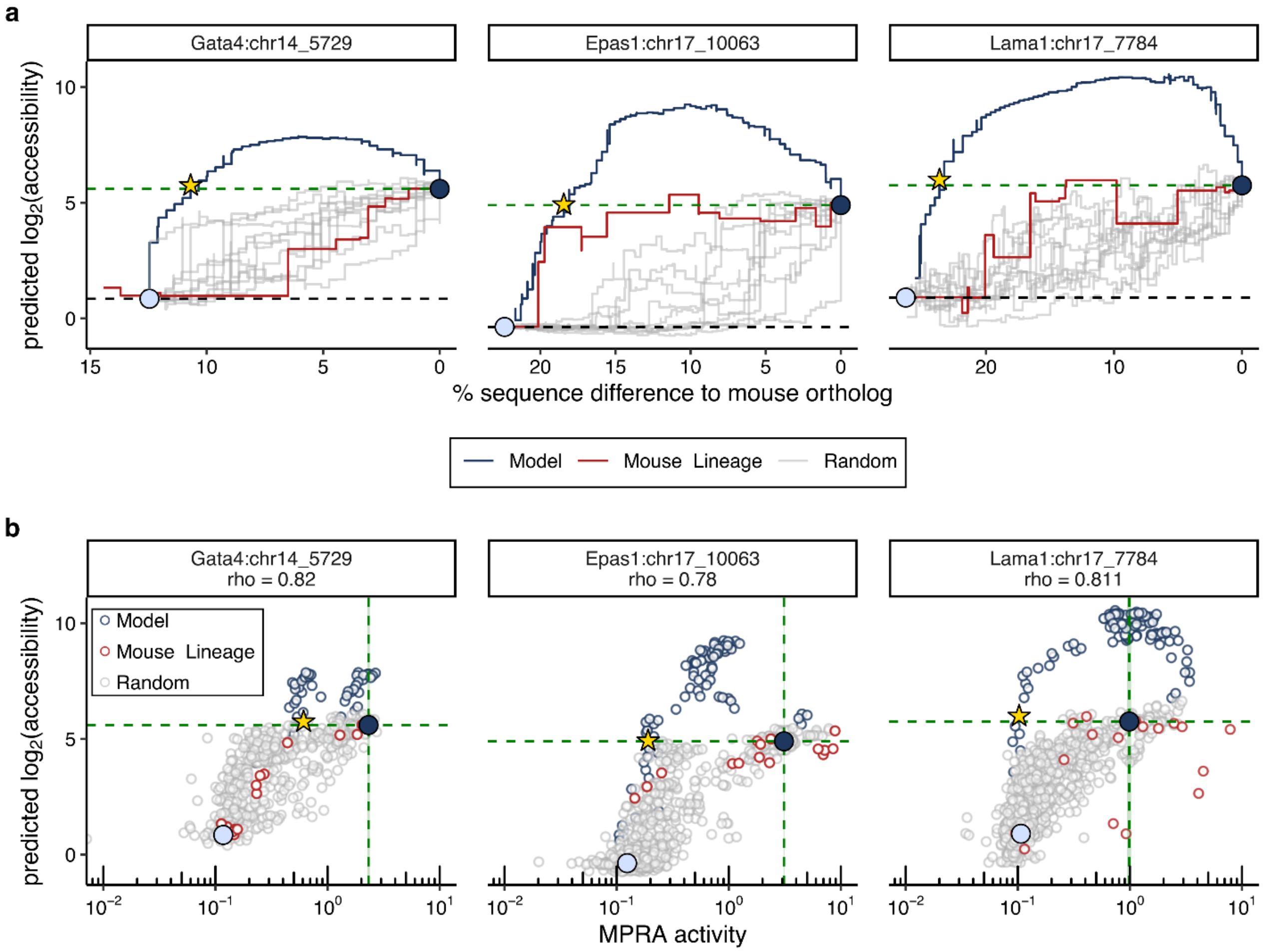


**Supplementary Figure 8**. **Comparison of model-predicted chromatin accessibility and experimentally measured enhancer activity during synthetic reconstitution. a)** Model predicted $\log_2$ accessibility (y-axis) versus sequence divergence (x-axis) for the reconstitution trajectories and the mouse evolutionary lineage of the *Gata4*, *Epas1*, and *Lama1* CREs (from top to bottom). Model-optimized, random-order, and phylogeny-inferred evolutionary trajectories are shown in navy, gray, and red, respectively. Green and gray horizontal dotted lines indicate the predicted accessibility of the extant mouse ortholog and the inferred common mammalian ancestral ortholog, respectively. **b)** Scatterplots comparing MPRA activity (x-axis) and ChromBPNet-predicted log2 accessibility (y-axis) for all intermediate sequences across reconstitution trajectories. Points are colored by trajectory type (model-optimized, navy; random, gray; evolutionary lineage, red). Spearman correlation coefficients are shown for each CRE at the top. Large circles denote the initial (light blue) and final (navy) intermediates for all trajectories, and stars denote the earliest intermediate reaching or exceeding the predicted accessibility of the endogenous mouse CRE.

bioRxiv preprint doi: https://doi.org/10.64898/2026.04.20.719714; this version posted April 21, 2026. The copyright holder for this preprint (which was not certified by peer review) is the author/funder, who has granted bioRxiv a license to display the preprint in perpetuity. It is made available under aCC-BY 4.0 International license.

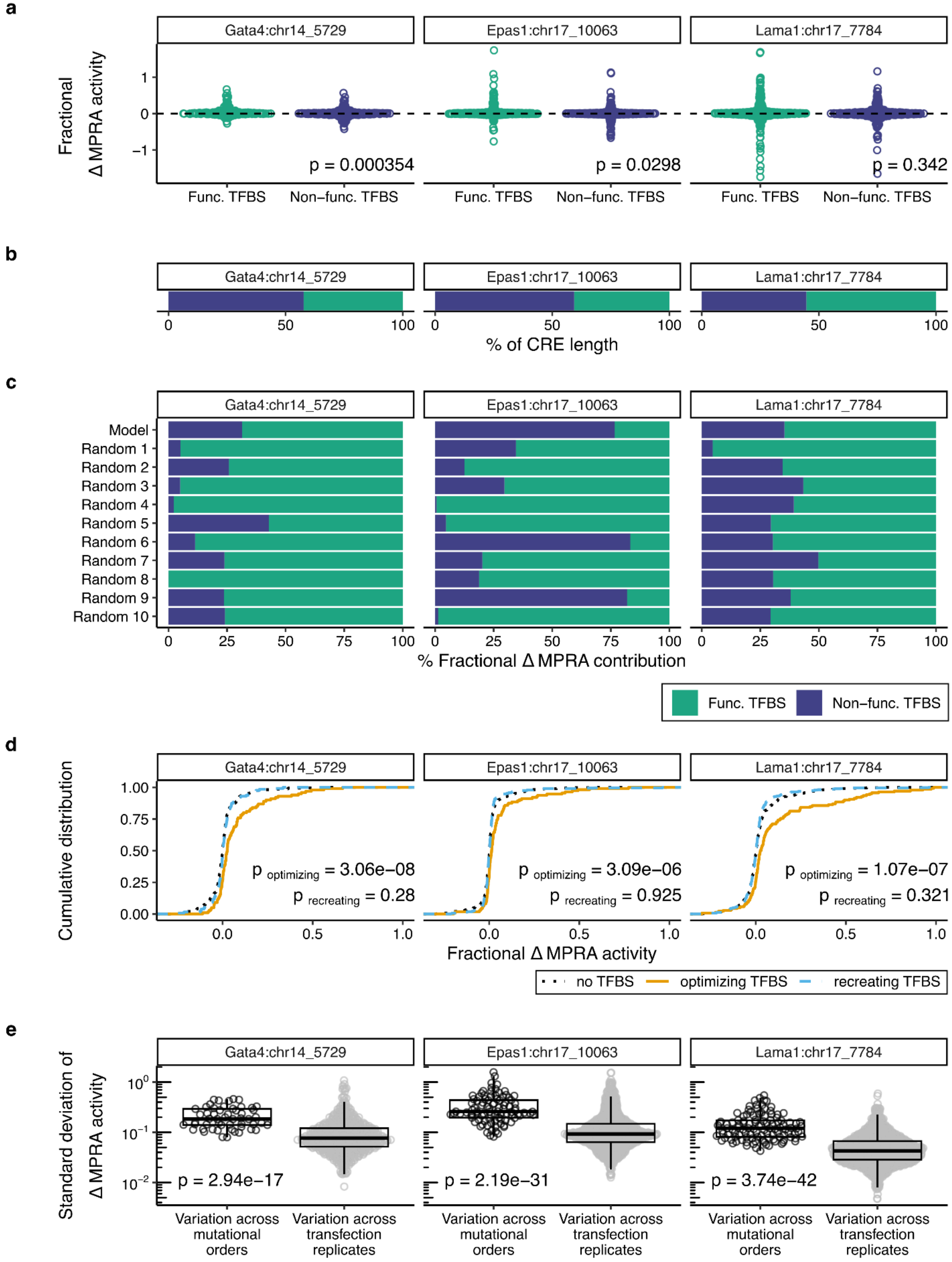


**Supplementary Figure 9**. **Contribution of TFBS-associated mutations and epistasis to enhancer reconstitution trajectories. a)** Fractional changes in MPRA activity associated with individual mutations across all reconstitution trajectories, stratified into mutations overlapping functional TFBS (green) or non-TFBS (purple) positions, in the extant mouse CRE. P-values report Wilcoxon rank-sum tests comparing TFBS-overlapping and non-TFBS mutations for each

bioRxiv preprint doi: https://doi.org/10.64898/2026.04.20.719714; this version posted April 21, 2026. The copyright holder for this preprint (which was not certified by peer review) is the author/funder, who has granted bioRxiv a license to display the preprint in perpetuity. It is made available under aCC-BY 4.0 International license.

CRE. **b)** Overall fraction of each CRE sequence overlapping functional TFBSs versus non-TFBS positions. **c)** Proportion of total fractional MPRA activity recovery attributable to mutations overlapping functional TFBSs (green) or non-TFBS (purple) positions, across all reconstitution trajectories for each CRE. **d)** Cumulative distributions of fractional MPRA activity changes grouped by mutation class: non-TFBS positions (black, dotted), TFBS-recreating mutations (cyan, dashed), and TFBS affinity-optimizing mutations (gold, solid). P-values indicate Wilcoxon rank-sum tests comparing TFBS-recreating or TFBS-optimizing mutations against mutations at non-TFBS positions, computed separately for each CRE. **e)** Comparison of order-dependent variability in mutational effects to experimental noise. Shown is the standard deviation of MPRA activity changes for each mutation across reconstitution trajectories (black; n = 11 trajectories) and across biological replicates within a trajectory (gray; n = 6 biological replicates per trajectory). Each point represents a single mutation. Box-and-whisker plots indicate the median, interquartile range, and whiskers extending to 1.5× IQR. P-values report Wilcoxon rank-sum tests comparing trajectory-level and replicate-level variability for each CRE.

bioRxiv preprint doi: https://doi.org/10.64898/2026.04.20.719714; this version posted April 21, 2026. The copyright holder for this preprint (which was not certified by peer review) is the author/funder, who has granted bioRxiv a license to display the preprint in perpetuity. It is made available under aCC-BY 4.0 International license.

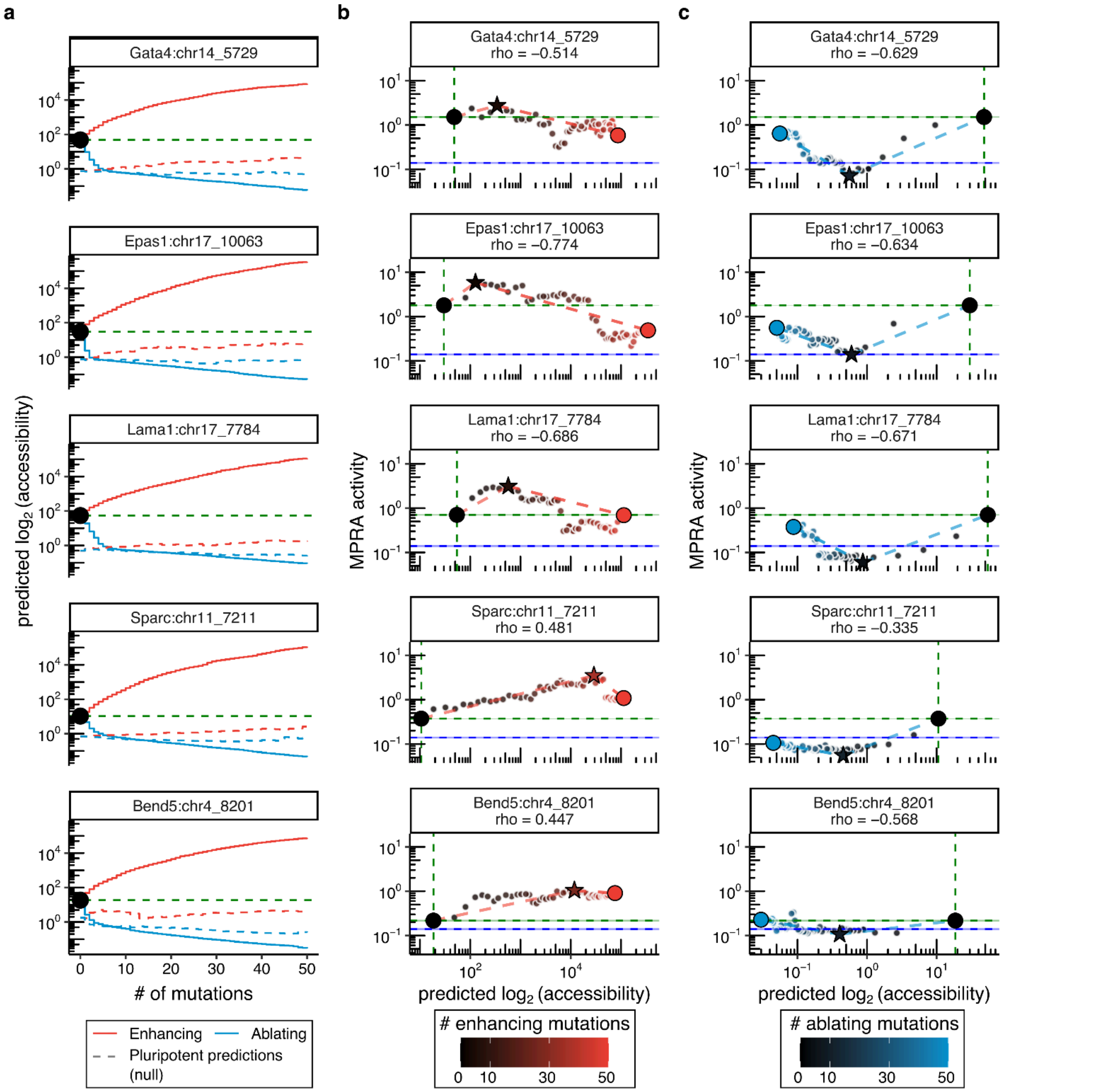


**Supplementary Figure 10. Comparison of predictions vs. observations upon model-guided enhancement or ablation of activity of endogenous parietal endoderm CREs. a)** ChromBPNet-predicted $\log_2$ chromatin accessibility (y-axis) across sequential gradient steps (x-axis) for model-guided modulation trajectories of the *Gata4*, *Epas1*, *Lama1*, *Sparc*, and *Bend5* CREs (top to bottom). Trajectories optimized for enhancement (red) or ablation (blue) are shown. Black circles denote the predicted accessibility of the endogenous mouse sequence, and green dotted lines indicate the same reference level across steps. Dashed lines show predictions for the same sequences obtained using a null model trained on pluripotent chromatin accessibility. **b)** Scatter plots comparing ChromBPNet-predicted $\log_2$ accessibility (x-axis) to MPRA-measured activity (y-axis) for the enhancement (left) and ablation (right) trajectories. Points correspond to intermediate sequences along each trajectory; the starting sequence (black circle), best-performing intermediate for the corresponding objective (star), and final intermediate (red or blue circle) are indicated, and the color gradient denotes the number of mutations introduced. Horizontal dotted lines (with shading) denote the activity of the endogenous mouse CRE (green) and the minimal-promoter (minP) background control (blue). Vertical green dotted lines denote the predicted accessibility of the endogenous mouse CRE. Spearman correlation coefficients (ρ) between predicted accessibility and MPRA activity are shown for each combination of CRE and training objective.

bioRxiv preprint doi: https://doi.org/10.64898/2026.04.20.719714; this version posted April 21, 2026. The copyright holder for this preprint (which was not certified by peer review) is the author/funder, who has granted bioRxiv a license to display the preprint in perpetuity. It is made available under aCC-BY 4.0 International license.

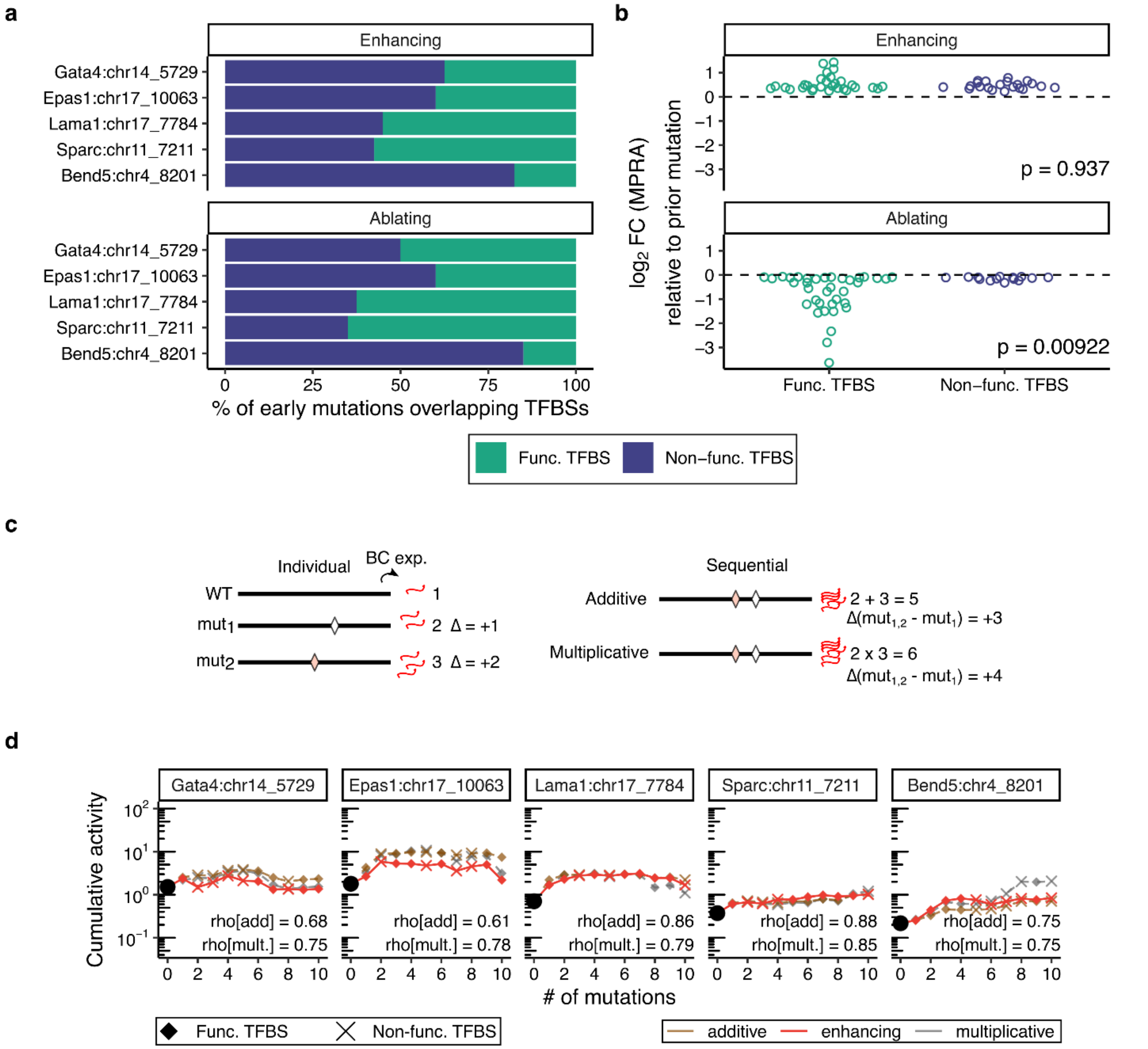


**Supplementary Figure 11. Preferential targeting of functional TFBSs and epistasis during model-guided modulation of parietal endoderm CREs. a)** Composition of the first ten model-nominated mutations for each CRE under the enhancement and ablation objectives, stratified by whether mutations overlap functional TFBSs (green) or non-TFBS positions (purple) in the extant mouse sequence. **b)** $Log_2$ fold-changes in MPRA activity contributed by each of the first ten mutations, measured relative to the immediately preceding intermediate sequence, shown separately for enhancement and ablation objectives and stratified by mutations overlapping functional TFBSs (green) or non-TFBS positions (purple). P-values indicate Wilcoxon rank-sum tests comparing activity changes between TFBS-overlapping and non-TFBS mutations. **c)** Schematic of the additive and multiplicative models used to estimate the combined effects of successive mutations. In the additive model, mutational effects on reporter output are assumed to combine additively, whereas in the multiplicative model they are assumed to combine multiplicatively. **d)** Cumulative MPRA activity for the first ten mutations for each of the five CREs, compared to expectations from the additive (brown dashed) and multiplicative (grey dashed) models. MPRA-measured activity is shown as solid red lines. Error bars denote the standard deviation across three biological replicates. The black circle marks the activity of the endogenous mouse CRE. Symbols indicate whether individual mutations overlap functional TFBSs (diamonds) or non-TFBS positions (crosses). Spearman correlation coefficients (ρ) between observed and expected activities are shown for each CRE and model.

bioRxiv preprint doi: https://doi.org/10.64898/2026.04.20.719714; this version posted April 21, 2026. The copyright holder for this preprint (which was not certified by peer review) is the author/funder, who has granted bioRxiv a license to display the preprint in perpetuity. It is made available under aCC-BY 4.0 International license.

## SUPPLEMENTARY NOTE

**Supplementary Note 1. Comparison of four sequence-based modeling strategies.** To assess how well enhancer activity across the mammalian phylogeny can be predicted directly from sequence, we evaluated four sequence-based modeling strategies of increasing complexity across five parietal endoderm CREs, including: **(i)** a simple "functional motif count", obtained by projecting TFBSs experimentally mapped in extant mouse onto orthologs; **(ii)** a generalized linear model (GLM) trained on predicted TF binding affinities; **(iii)** a gapped k-mer support vector machine model, gkm-SVM[45], trained on parietal endoderm accessibility; and **(iv)** a deep convolutional neural network (CNN), ChromBPNet[46], trained on the same accessibility profiles. Evaluating these models in parallel allowed us to assess how much of the striking functional diversity observed across orthologs of each CRE can be explained by progressively richer representations of sequence information.

Functional motif counts: As a baseline approach, we projected mouse TFBSs mapped by saturation mutagenesis[38] onto all orthologs (7-15 mouse TFBSs per CRE, and counted those retaining comparable or greater predicted affinity. Most orthologs contained fewer sites than the mouse reference, and the number of sites was moderately correlated with MPRA activity (ρ = 0.15-0.78). However, the impact of site loss differed by element: *Gata4* CRE orthologs generally required near-complete retention of equivalent TFBSs to maintain activity, while *Epas1* and *Lama1* CRE orthologs often preserved or even exceeded mouse activity despite losing multiple mouse motifs (**Extended Data Fig. 1a**) These results indicate that simple motif composition partly explains cross-species functional variation, and suggests substantial heterogeneity in the rigidity versus flexibility across elements.

GLMs based on predicted TF binding affinity: To incorporate quantitative motif strength, we fit a GLM for each CRE using summed predicted affinities for key parietal endoderm TFs as predictors. Relative to simple counts, these models roughly doubled correlation with MPRA activity for 3 of the 5 CREs (ρ = 0.30-0.61; **Extended Data Fig. 1b**), indicating that variation in TFBS affinity also contributes meaningfully to functional divergence across orthologs. Consistent with saturation mutagenesis data, AP-1 affinity most strongly predicted activity for *Epas1* CRE orthologs, while *Sox17* affinity best explained variation among *Gata4* CRE orthologs (**Extended Data Fig. 1c**).

gkm-SVM trained on chromatin accessibility: As a third approach, we trained a model using gkm-SVM[45], a support vector machine framework that uses gapped k-mers, on pseudobulk parietal endoderm ATAC-seq peaks from our previous scATAC-seq dataset[34]. Unlike the motif-based models above, gkm-SVM is trained to predict chromatin accessibility, rather than enhancer activity, directly from sequence. When applied to evolutionary and ancestral CRE orthologs, gkm-SVM predictions showed weak to moderate correlations with MPRA activity in four of five cases (ρ = 0.01–0.57; **Extended Data Fig. 1d**). Notably, gkm-SVM outperformed motif count and GLM approaches for the *Lama1* (ρ = 0.57) and *Bend5* (ρ = 0.41) CREs, indicating that k-mer–based sequence features capture aspects of regulatory variation not represented by motif-centric models.

ChromBPNet modeling of chromatin accessibility: To leverage recent advances in deep learning-based modeling of regulatory grammar, we applied ChromBPNet[46], a CNN for modeling chromatin accessibility, trained on the same pseudobulked parietal endoderm scATAC-seq data as the gkm-SVM model parietal endoderm, as well as data from other mEB-derived lineages (**Extended Data Fig. 2a**). Predicted and observed accessibility were well-correlated for each pluripotent and germ layer lineages, both on consensus peaks (ρ = 0.60-0.83) and differential accessible peaks (ρ = 0.61-0.78) (**Extended Data Fig. 3a-b**). Moreover, ChromBPNet predicted accessibility well over genomic regions corresponding to 4 of the 5 CREs tested (all but the *Sparc* CRE; **Extended Data Fig. 3c**). Despite being a predictor of accessibility, ChromBPNet performed

bioRxiv preprint doi: https://doi.org/10.64898/2026.04.20.719714; this version posted April 21, 2026. The copyright holder for this preprint (which was not certified by peer review) is the author/funder, who has granted bioRxiv a license to display the preprint in perpetuity. It is made available under aCC-BY 4.0 International license.

surprisingly well when as a predictor of MPRA activity (average of accessibility profile across embedding in 100 random sequences), (ρ = 0.35-0.64; **Extended Data Fig. 3d**).

Comparative performance of sequence models: While all four approaches recovered meaningful sequence–function relationships, ChromBPNet provided the most consistent and generalizable performance across CREs on our task. Motif counts provide an intuitive baseline but are overly reductionist, collapsing each ortholog to the presence or absence of a small set of mouse-mapped TFBSs, missing syntax, compensatory changes, and gains of novel motifs. GLMs improved predictions for several CREs, but are trained "per CRE" and thus do not generalize to new elements and are further limited by linear assumptions. gkm-SVM, trained once on genome-wide accessibility, avoids CRE-specific overfitting but exhibits variable performance across elements. In contrast, ChromBPNet achieved moderately strong correlations across all five CREs, offering the most stable and generalizable sequence-to-activity predictions. This, coupled with its compatibility with nucleotide-resolution interpretation tools, motivated us to use it as our primary model for downstream analyses.

Base-resolution interpretation and motif perturbation analyses: We also leveraged ChromBPNet's base-resolution interpretations to query what sequence features drive accessibility across species. Comparing mouse and rat *Gata4* CREs, the model highlighted the loss of the *Sox17* motif in rat (**Extended Data Fig. 2c**), a site gained along the mouse lineage after divergence from Anc38 (**Fig. 2f-g**; **Supplementary Fig. 5a**) and coinciding with gain of function as measured by MPRA. Similarly, model interpretations for the mouse and African wild dog *Epas1* CREs emphasized the conserved Foxa2-Sox17-Gata4/6 triplet as the dominant contributor to predicted accessibility in both species (**Extended Data Fig. 2d**). ChromBPNet also flagged specific substitutions within this module that matched effects measured by saturation mutagenesis in mouse, including their impacts on predicted TFBS affinity (**Extended Data Fig. 2e**). In the African wild dog ortholog, these substitutions decreased predicted Foxa2 and Gata4/6 affinity (3.6-fold and 3.7-fold, respectively) while increasing Sox17 affinity 2.3-fold, potentially examples of buffering via compensatory changes.

Finally, we performed *in silico* insertions of key parietal endoderm TF motifs into non-accessible background sequences, and evaluated their impact on model-predicted chromatin accessibility. Particularly for Fox, Sox and Gata motifs, *in silico* insertion yielded "peak gains" with the parietal endoderm model but not other germ layer models (**Extended Data Fig. 4a**). Furthermore, analysis of learned motifs using TF-MoDISco[83] revealed high concordance between models trained on pseudobulk parietal endoderm scATAC-seq and PYS-2 bulk ATAC-seq data (**Extended Data Fig. 4b**).

These complementary evaluators of sequence syntax highlight how diverse regulatory architectures can support transcriptional activity in PYS-2 cells. Elements such as the *Gata4* CRE appear to rely on a precise grammar structure that constrains sequence evolution, whereas others, like *Epas1*, reach activity once a threshold of overall binding affinity is reached—a pattern consistent with a billboard-like regulatory model[84]. Although chromatin accessibility alone does not determine enhancer activity, it remains a prerequisite for regulatory potential[85], providing a useful surrogate for modeling sequence features associated with activation. Accordingly, we used the accessibility-trained model to identify and quantify base-resolution contributions to transcriptional activity in the analyses that follow.
